# Cell Therapy With Human ESC-Derived Cardiac Cells: Clinical Perspectives

**DOI:** 10.3389/fbioe.2020.601560

**Published:** 2020-10-26

**Authors:** Philippe Menasché

**Affiliations:** ^1^Department of Cardiovascular Surgery, Hôpital Européen Georges Pompidou, Paris, France; ^2^PARCC, INSERM, University of Paris, Paris, France

**Keywords:** embryonic stem cell, cardiac cell, heart failure, ischemic heart, induced pluripoten stem cell

## Abstract

In the ongoing quest for the “ideal” cell type for heart repair, pluripotent stem cells (PSC) derived from either embryonic or reprogrammed somatic cells have emerged as attractive candidates because of their unique ability to give rise to lineage-specific cells and to transplant them at the desired stage of differentiation. The technical obstacles which have initially hindered their clinical use have now been largely overcome and several trials are under way which encompass several different diseases, including heart failure. So far, there have been no safety warning but it is still too early to draw definite conclusions regarding efficacy. In parallel, mechanistic studies suggest that the primary objective of “remuscularizing” the heart with PSC-derived cardiac cells can be challenged by their alternate use as *ex vivo* sources of a biologically active extracellular vesicle-enriched secretome equally able to improve heart function through harnessing endogenous repair pathways. The exclusive use of this secretome would combine the advantages of a large-scale production more akin to that of a biological medication, the likely avoidance of cell-associated immune and tumorigenicity risks and the possibility of intravenous infusions compatible with repeated dosing.

## Why Pluripotent Stem Cells?

### Rationale for the Use of Cardiac-Committed Cells

So far, it is fair to acknowledge that no one can claim that a given cell type has unequivocally demonstrated its superiority over another for inducing heart repair and the attendant improvement in left ventricular function. Nevertheless, the few comparative experimental studies which have been published suggest the interest of using cells which are phenotypically matched to those of the organ to be repaired and thus highlight the potential benefits of cardiac-committed cells. A first comparison of human induced pluripotent stem cell (iPSC)-derived cardiomyocytes versus human mesenchymal stromal cells (MSC) injected 30 min after permanent coronary artery ligation in rats reported that the former tended to improve left ventricular function to a greater extent and significantly reduced fibrosis compared with MSC (Citro et al., 2014). In a subsequent head-to-head comparison of different human stem cell types, cardiosphere-derived cells were found superior to MSC from bone marrow and adipose tissue and to bone marrow-derived mononuclear cells (BMMNCs) for improving post-infarction cardiac function and cell engraftment in a mouse model also treated just after ligation of the left anterior descending artery (Li et al., 2012). Likewise, human embryonic stem cell (ESC)-derived cardiomyocytes and mesodermal cardiovascular progenitors were found to similarly improve post-infarction systolic function in contrast to BMMNCs in rat hearts treated 4 days after a 60 min period of coronary artery occlusion (Fernandes et al., 2015). In keeping with these data, the more recent pig study of Ishida et al. (2019) shows that in comparison of skeletal myoblasts and MSC, iPSC-derived cardiomyocytes (all cells being of human origin) yielded the best outcomes with regard to regional function, oxidative metabolism, vascular density and limitation of apoptosis. However, an opposite conclusion emerged from another comparative study of iPS derivatives where MSC were found superior to cardiomyocytes for the improvement of cardiac function in heart failure, an effect primarily attributed to their immunomodulatory effects (Liao et al., 2019). This discrepancy could be related to very specific features of this protocol: the induction of heart failure by coronary artery occlusion associated with rapid pacing, a setting known for its instability and the intensity of neuro-hormonal stimulation (Yarbrough, 2003); the use of different PSC sources (ESC for cardiomyocytes and iPSC for MSC); the application of a uniform immunosuppression regimen to MSC and cardiomyocytes whereas the former are known to be more immune evasive, which was indeed reflected in that study by their lower expression of Human Leukocyte Antigen (HLA)-II; the greater susceptibility of ESC-derived cardiomyocytes to an immune response could thus have accounted for their lower rate of survival and their (slightly) inferior functional performance. In brief, these data are difficult to interpret because of all these confounders and do not challenge the concept that an optimal therapeutic benefit likely requires that both transplanted and host cells belong to the same lineage.

In practice, adult tissue sources of cardiac cells are limited to cardiospheres and c-*kit*^+^ cardiac stem cells (CSC) whose harvest from heart tissue does not equate to their cardiac lineage commitment. Cardiospheres are agglomerates of several cell types, predominantly MSC, harvested from the right ventricle by an endomyocardial biopsy (Smith et al., 2007) with a subsequent intracoronary delivery while c-*kit*^+^ CSC are grown from a right appendage biopsy taken during a coronary artery bypass operation before being also reinjected into the coronary arteries. However, the first have failed to show benefits in an ischemic cardiomyopathy phase II trial which was prematurely interrupted in April, 2017 for futility; however, the use of these cells in Duchenne muscular dystrophy has yielded an encouraging efficacy signal (Taylor et al., 2019) which needs to be confirmed by the ongoing HOPE-II study, planned to randomize 84 non-ambulatory and ambulatory patients with Duchenne muscular dystrophy to intravenous infusions of either 150 million cardiosphere-derived cells every 3 months for a total of 4 doses or placebo. The use of c-*kit*^+^ CSC has been largely based on preclinical data now considered to be in part fraudulent^1^ and there is some consensus that these cells are rather endowed with an angiogenic potential (van Berlo et al., 2014). Four trials are currently registered in the ClinicalTrials.gov website, of which one (CONCERT-HF) which has tested transendocardial injections of autologous MSC, c-*kit*^+^ CSC, alone or in combination, in patients with ischemic heart failure, was temporarily paused in October, 2018. Among the remaining 3 studies, only one, the CHILD trial, which assesses the effects of intramyocardial injection of autologous c-*kit*^+^ cells in 32 patients with hypoplastic left heart syndrome, is currently recruiting. A second one (TAC-HF-II) planned to test transendocardial injection of autologous MSC alone or in combination with CSC in 55 patients with ischemic left ventricular dysfunction, has not yet started recruiting. The third trial (JOKER) was designed to assess the effects an intracoronary infusion of autologous c-*kit*^+^ cells expanded from a right appendage biopsy taken during coronary surgery in a small group of six patients still presenting a left ventricular ejection fraction < 40% after their revascularization; it is reported active but not recruiting. Outcomes of these trials will hopefully shed some light on the place of these cells in the context of cell-based heart repair.

### Interest of PSC as a Source of Cardiac-Committed Cells

Given the limitations of these adult sources of cardiac-committed cells, it has looked sound to consider the alternate use of pluripotent stem cells (PSC) to leverage their intrinsic ability to generate lineage-specific cells in response to appropriate cues and thus coax them toward a cardiac differentiation pathway. Since the seminal work of Caspi et al. (2007) showing that ESC-derived cardiomyocytes improved myocardial performance in chronically infarcted rats, a flurry of studies have confirmed the functional efficacy of PSC cardiac derivatives. This has been a strong incentive for developing multiple and various techniques for PSC scale-up and differentiation, the detailed description of which is beyond the scope of this article (for a recent review, see Le and Hasegawa, 2019). Enough is to say that the extensive documentation on the raw products required for a regulatory approval makes highly desirable to use the most straightforward and cost-effective procedures, which has guided our choice of an only two cytokine-based technique described below. Another advantage of PSC is their scalability which is critical particularly if the objective is the “remuscularization” of extensively scarred post-infarction myocardial areas (as discussed below). One could argue that this property is shared by MSC but it is not totally true as there is some evidence that increasing the number of MSC passages can shift their phenotype toward an aging pattern translating into an impaired functionality of the cells (Yin et al., 2019).

The ability to control the differentiation pathway of PSC gives the flexibility of “freezing” it at the desired stage and thus provides the option of transplanting early progenitor cells or more mature cardiomyocytes. Each of these cell types has advantages and inconvenient. Early progenitors feature a greater plasticity which could allow them to differentiate in both cardiac and vascular cells and their predominant reliance on anaerobic glycolysis might enhance their survival in a poorly oxygenated environment. This assumption is supported by the findings of Halbach et al. (2014) that the highest persistence and grade of electrical coupling of intramyocardially transplanted fetal cardiomyocytes from different developmental stages is achieved by intermediate cells (days 14.5) compared with earlier and later stages (days 9.5 and 18.5, respectively). An additional feature of early progenitor cells is their greater secretory profile (Agarwal et al., 2017; El Harane et al., 2018), which can be an advantage if one relies on a predominant paracrine mechanism of action (see below). At the opposite, an early progenitor cell population can still be “contaminated” by pacemaker cells behaving as foci of automaticity and thus predisposing to arrhythmias (Ichimura et al., 2020). Conversely, this issue is addressed by the use of more mature cardiomyocytes which are thought to be endowed with a greater force-generating potential but, in turn, may be more susceptible to death once transplanted in hypovascularized areas because of their reliance on oxidative phosphorylation. At the end, it is fair to admit that currently the few studies which have compared different stage-specific cardiac differentiated cells have failed to provide unequivocal evidence for the superiority of one type over the other (Fernandes et al., 2015; Ye et al., 2015).

### The ESCORT Trial

**FIGURE 1:**
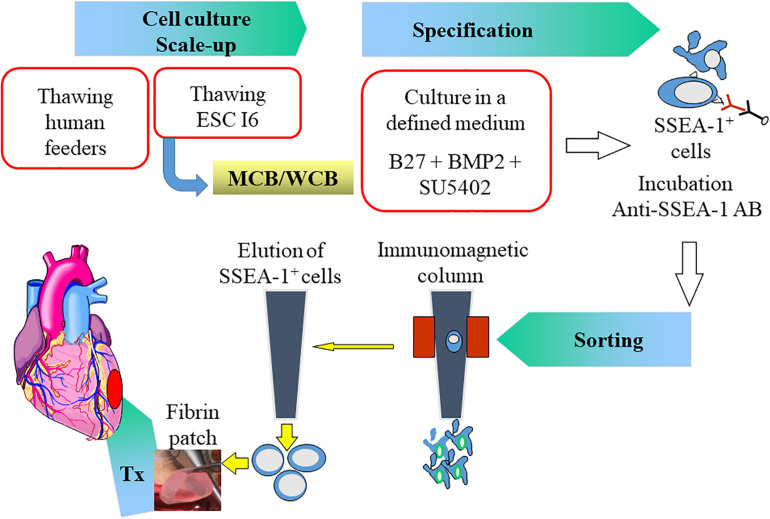
Summary of the protocol in the ESCORT trial. Human Embryonic Stem Cells (ESC) from the I6 cell line were expanded on human feeders to generate a Master/Working Cell Bank (MCB/WCB). Expanded pluripotent stem cells (scale-up) were then cardiac-committed (specification) by a 4-day exposure to Bone Morphogenetic Protein (BMP)-2 and a Fibroblast Growth Factor inhibitor (SU5402) in B27 medium. Committed cells express the Stage-Specific Embryonic Antigen (SSEA)-1 indicating their loss of pluripotency and could thus be immune-magnetically sorted using an anti SSEA-1 antibody. The SSEA-1 enriched cardiovascular progenitor cell population was then embedded in a fibrin patch which was transplanted onto the epicardium of the infarct area. AB: antibody; Tx: transplantation.

**FIGURE 2:**
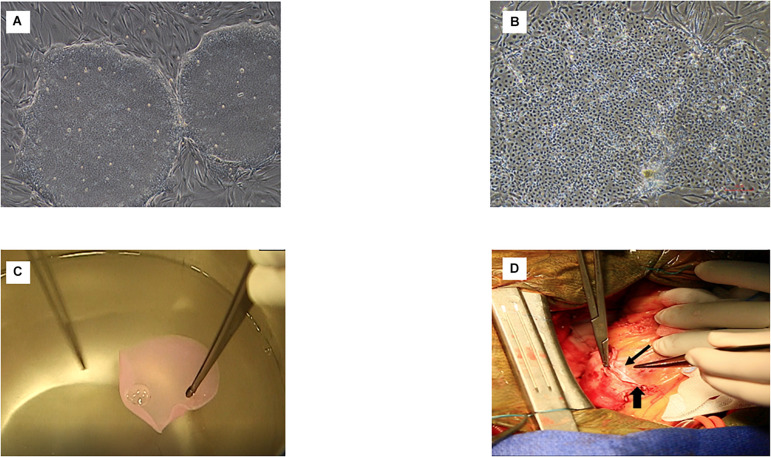
Main steps of the procedure in the ESCORT trial. **(A)** Pluripotent ESC of the I6 cell line. **(B)** Cardiovascular progenitors at the completion of the 4-day specification step. **(C)** Fibrin patch loaded with the cardiovascular progenitors (intra-operative picture showing the rinsing of the patch before its implantation in the patient). **(D)** Final step: the cell-loaded patch has been delivered onto the epicardium of the infarct area and is partly covered by a pericardial flap already sutured along one-half the infarct circumference, thereby creating a pocket (between the flap and the epicardium) inside which the patch has been slid; the long and thin arrow indicates the border of the patch. The short and wider arrow indicates the suture line of the pericardial flap to the epicardium. Once the cell-loaded fibrin patch seats within the pocket, this suture line will be completed along the remaining one-half of the infarct circumference to enclose it completely, thereby ensuring its stability while providing some trophic support to the underlying fibrin patch.

Our choice has been to transplant early progenitors differentiated from ESC in the ESCORT trial (NCT02057900) which was a first-in-man safety study of 6 patients with severe ischemic left ventricular dysfunction (median left ventricular ejection fraction: 26%; IQR: 22–32%) and in whom the cell therapy treatment was combined with a coronary artery bypass (Menasché et al., 2018). The trial was based on 10 years of preclinical studies in rodents and non-human primates (Bellamy et al., 2015; Menasché et al., 2015). Pluripotent ESC were first scaled-up to generate a master/working cell bank from which cells were collected, thawed, expanded in a defined medium on clinical-grade feeder cells (irradiated human foreskin fibroblasts) and then committed toward a mesodermal-cardiac lineage by a 4-day exposure to two cytokines (bone morphogenetic protein-2 and a FGF inhibitor) according to Good Manufacturing Practice (GMP) standards. As roughly 44% of the cells only responded to the cardio-instructive cues, a purification step was mandatory. The expression of stage-specific embryonic antigen (SSEA)-1, was then taken as a marker for loss of pluripotency and immunomagnetic sorting using a microbead-coupled anti-SSEA-1 antibody was used for selecting the committed cells. This resulted in the harvest of a highly purified population of SSEA-1^+^ cells characterized by a knock-down of the pluripotency gene *Nanog* and a parallel upregulation of the cardiac transcription factor *Isl-1*. These two markers, assessed by qPCR, were among the release criteria, with thresholds set as <0.1% and > 5%, respectively, expressed as fold changes relative to the undifferentiated population. Additional lot release criteria included cell viability and purity, with thresholds set at 90 and 95%, respectively, which were fully met for all the patients [median viability and purity rates of 96% (IQR: 96–96%) and 97.5% (IQR: 95.5–98.7%), respectively]. SSEA-1^+^ progenitor cells were then embedded into a fibrin patch generated by first mixing them with fibrinogen followed by addition of thrombin to induce rapid polymerization of the gel (fibrinogen and thrombin were components of a clinically used surgical glue). Finally, during the surgical procedure, a 20 cm^2^ piece of autologous pericardium was harvested and sutured to the epicardium along one-half the borders of the infarct area, thereby creating a pocket into which the cell-loaded fibrin patch was simply slipped. The free edge of the pericardial flap was then stitched to the remaining half of the infarct circumference, thereby enclosing the fibrin patch and ensuring its stability. The use of the pericardium was based on the assumption that it could act as a natural bioreactor providing growth factors to the underlying cellular graft and thus contributing to enhance early cell survival (Figures 1, 2). A median dose of SSEA-1^+^ *Isl*-1 cardiovascular progenitors of 8.2 million (IQR: 5–10 million) was delivered without any adverse intraoperative events. The primary end point was safety at 1 year, primarily assessed on (1) cardiac teratoma or remote tumor tracked by whole body computed tomography and fluorine-18 deoxyglucose positron emission tomography scans, (2) arrhythmias, detected by serial interrogations of the cardioverter-defibrillator implanted in all patients, and (3) alloimmunization, assessed by the presence of donor-specific antibodies. All patients had an uneventful post-operative course, except for one who died shortly after the operation from multiple comorbidities. With a maximum follow-up of 6 years, no patient presented an adverse event that could be related to the cells and/or the patch. While it would be meaningless to draw conclusions regarding efficacy, enough is to say that an encouraging signal was provided by a significant improvement of the wall motion of the cell/patch-treated segments during follow-up with a score that decreased from 4.2 ± 0.8 at baseline to 2.5 ± 0.4 at 1 year (*p* = 0.004 by the mixed model ANOVA on ranks). Of note, in 3 of the 4 patients who contributed these 1-year data, the treated segments had not been revascularized but, again, this cannot be taken as definite proof of efficacy because of the confounding effect of concomitant revascularization. Since we were not expecting a long-term cellular engraftment and primarily relied on a paracrine mechanism of action (see below), patients were only immunosuppressed transiently and while the initial planning was to give the drugs for 2 months, the duration was shortened to 1 month from the second patient onward. Drugs were given at a relatively low dosing (target trough levels of cyclosporine: 100–150 ng/ml; mycophenolate mofetil, 2 g/day) since our pre-operative mixed lymphocyte reaction assays had shown that SSEA-1^+^ cells are weakly immunogenic.

### Other PSC Clinical Trials

Other investigators have made the different choice of transplanting PSC-derived cardiomyocytes at a later stage of differentiation (although their persistent fetal-like phenotype precludes their assimilation to *bona fide* myocardium-resident cardiomyocytes) and have switched to iPSC as the source cells for practicality and/or ethical reasons. Once differentiated, iPSC-derived cardiomyocytes share with ESC the ability to improve the function of infarcted hearts (Lee et al., 2017) but also the lack of long term engraftment (Okano and Shiba, 2019). The use of iPSC has been aggressively promoted by those who oppose ESC for religious reasons with the premise that they could be differentiated from the patient’s own somatic cells, thereby obviating the use of immunosuppression. This argument is no longer tenable since there is a consensus that iPSC for clinical purposes should rather be harvested from healthy donors, i.e., in an allogeneic mode, to improve safety and potency and decrease costs. This is actually the case for two trials in patients with ischemic heart failure: one in Japan aims at grafting *allogeneic* iPSC-derived cardiomyocytes in 10 patients with a severe cardiomyopathy under the form of cell sheets prepared according to a well-documented technology (Guo et al., 2020). The announcement of the first operation in this series has been made in the lay press in January, 2020^2^, but at the time of this writing, this trial is not registered on University Hospital Medical Information Network (UMIN CTR), Japan^3^ and only its 3-patient roll-in phase is listed as terminated (UMIN000032989). The second trial which is in preparation in Germany plans to graft engineered heart constructs (Tiburcy et al., 2017) made of iPSC-derived cardiomyocytes and collagen in patients with end-stage heart failure. Of note, in these two studies, cell therapy will be used as a stand-alone procedure which will allow a true assessment of its effects without the confounding effect of an associated procedure and also makes possible a less invasive approach by a small left thoracotomy. Conversely, another trial (HEAL-CHF, NCT03763136), in China, should include five participants with ejection fractions between 30 and 45% who will be injected intramyocardially with 100 million *allogeneic* iPSC-derived cardiomyocytes but in conjunction with coronary artery bypass grafting. This study is currently recruiting. A last trial planned to include 3 patients with chronic heart failure and in whom *autologous* iPSC-derived cardiomyocytes would be delivered intravenously has been registered in ClinicalTrials.gov in November 2018 (NCT03759405) but has surprisingly not yet started recruiting.

### The Issue of Dosing

A critically relevant issue raised by transplantation of PSC-derived cardiomyocytes, regardless of their ESC or iPSC source, is their optimal dosing. Several factors have to be taken into account, including the mass of lost myocardium, particularly if the objective is its remuscularization (as discussed below), the rate of cell attrition and the possible proliferation of the surviving cells although the latter factor is of unlikely clinical relevance because of the consistent finding of the absence of sustained cell engraftment. In a meta-analysis of stem cells in large animal studies (primarily performed in pigs), cells doses were extremely variable, ranging from 5 × 10^5^ to 25 × 10^6^ for c-*kit* CSC, from 1.3 × 10^6^ to 1 × 10^7^ for cardiospheres and also averaged 1 × 10^7^ for Sca-1 + CSC. Interestingly, in the only dose-escalating study of cardiospheres (Yee et al., 2014), the highest dose (150 million) was found superior to the lower ones when the assessment was made 4 weeks after treatment but the benefit on global and regional left ventricular function (versus a control group) was lost in a subsequent cohort receiving this high dose but assessed at a later time point (8 weeks). In a Phase II dose-escalation study of allogeneic mesenchymal precursor cells in patients with ischemic or non-ischemic heart failure (Perin et al., 2015), the best outcomes were yielded by the highest dose (150 million versus 25 and 75 million) and a similar dose-dependent response (better with 100 versus 20 million cells) has been reported in the TRIDENT trial in which allogeneic bone marrow-derived-MSC were delivered in patients with ischemic cardiomyopathy (Florea et al., 2017). These data are consistent with those of a metaanalysis of 914 MSC clinical trials (for all types of indications) in which the minimal effective dose ranged between 100 and 150 million cells (Kabat et al., 2020). Admittedly, however, none of these clinical studies have used PSC derivatives. Looking exclusively at those transplanted in non-human primate models generates conflicting results. Chong et al. (2014) reported an extensive remuscularization of the infarcted myocardium injected with 1 × 10^9^ ESC-derived cardiomyocytes but the small sample size precluded any meaningful functional assessment. Conversely, despite injecting a similar cell number, Romagnuolo et al. (2019) failed to show a benefit on infarct size or global left ventricular function (with no correlation between cardiomyocyte purity and graft size) after 4 weeks. In contrast, Liu Y.-W. et al. (2018) reported a functional benefit following transplantation of a slightly lower dose (7.5 × 10^8^) of ESC-derived cardiomyocytes and in the study of Shiba et al. (2016), a post-transplantation improvement of contractile function at 4 and 12 weeks could even be yielded by an almost twofold lower dose dose (4 × 10^8^ iPSC-derived cardiomyocytes). Finally, in another study using ESC-derived cardiovascular progenitors, a much lower dose (1 × 10^7^) also improved heart function provided the immunosuppression regimen was appropriate (Zhu et al., 2018). Put together, these data suggest that the highest doses are not necessarily the most effective, particularly in view of the risk of arrhythmias outlined in the next section, and although the issue of the optimal dosing is still open, they tend to support the earlier experimental findings that even though increasing the dose of ESC translates into an increased graft size, this may not reflected by an improvement in heart function (van Laake et al., 2009).

## How Do PSC-Derived Cardiac Cells Work?

### The “Remuscularization” Hypothesis

A logical objective of using PSC-derived cardiac cells is to structurally replace host cardiomyocytes which have been irreversibly damaged. This forms the basis of the “remuscularization” concept which entails the transplantation of cardiomyocytes, as mature as they can, or cardiac progenitors expected to complete their differentiation under the influence of local cues. The goal is that these cells stably engraft and seamlessly couple with host cardiomyocytes enabling the cellular graft to synchronously contribute to heart pump function while avoiding electrical instability. Several studies in both rodents and large animals, including non-human primates, have documented histologically the feasibility of generating large grafts recolonizing part of the scar tissue and predominantly made of cardiomyocytes, with evidence for their progressive maturation over time, an electrical coupling with host cardiomyocytes, as assessed by fluorescent calcium indicators (despite an often low connexin-43 immunoreactivity) and an associated improvement of left ventricular function of varying degree demonstrated by magnetic resonance imaging (Liu Y.-W. et al., 2018; Romagnuolo et al., 2019) or echocardiography and micro computerized tomography (Chong et al., 2014; Shiba et al., 2016). Despite being intellectually sound and attractive, this approach raises two major clinically relevant challenges: maintenance of long term cell survival and arrhythmias.

#### Cell Survival

If one targets the generation of a new myocardial tissue, it is expected that the grafted cells will remain engrafted and alive over time. Unfortunately, cells tend to die rapidly following transplantation, which has led to the investigation of multiple empowering strategies, primarily based on chemical or physical (heat shock) preconditioning and genetic engineering (Mohsin et al., 2011), but these approaches have been challenging to translate clinically, most likely because of their complexity, cost and difficulties to meet regulatory standards. In practice, three main factors of cell death have been identified. The first is the loss of cell anchorage to an extracellular matrix which occurs at the time of their usual dissociation before injection; this can be addressed by incorporation of the cells in a biomaterial which has a dual interest: (1) it provides a three-dimensional template conducive to extracellular matrix secretion enhancing cell cohesiveness, and (2) it acts as a shielding structure that increases cell retention. It is beyond the scope of this article to discuss the choice of biomaterials. Suffice is to say that if they are considered for catheter-based delivery, they should feature shear-thinning properties allowing their injection followed by an *in situ* gelation to improve retention of the matrix-encapsulated cells (Ban et al., 2014), whereas, they should have mechanical properties allowing to generate an easily manipulable patch if they are poised to an intraoperative epicardial application, as in the ESCORT trial and others. A second factor of cell death is the hostile nature of the environment they are implanted in, with a mix of inflammatory, hypovascularized and scarred areas which makes challenging for the transplanted cells to survive. One way of addressing this issue is to co-transplant them with supportive vascular and/or stromal cells to provide trophic and structural support. The critical role of non-cardiac cells (at a roughly 30% ratio) for increasing the therapeutic potential of iPSC-derived cardiomyocytes has been established (Iseoka et al., 2018) and further confirmed recently by the ability of ESC-derived epicardial cells co-transplanted with ESC-derived cardiomyocytes to enhance cardiac graft size and heart function in athymic rats treated 4 days after a 60 min coronary artery ligation (Bargehr et al., 2019). The study by Gao et al. (2018) is of even greater clinical relevance in that it used a large animal (porcine) model to establish the functional benefits of a composite patch construct made of iPSC-derived cardiomyocytes, endothelial and smooth muscle cells. In the clinics, these co-transplantation are technically doable as exemplified by the TAC-HF-II and the Japanese iPS sheet trials but they may complicate the cell manufacturing process. A third, and possible, the most challenging cause of cell death, is rejection of these allogeneic PSC derivatives. Currently, only drug-based immunosuppression is used to prevent rejection but despite the persisting uncertainties regarding the optimal drug regimen, it is admitted that these drugs are fraught with side-effects which may lead to their discontinuation with an attendant loss of the grafted cells (Guan et al., 2020). A variant of this approach could be a “biological” immunomodulation by co-transplantation of MSC which have been shown, at least in a subcutaneous implantation model, to control allogeneic iPSC-CM rejection via regulatory T cells and cell-cell contact with activated lymphocytes (Yoshida et al., 2020), however, because MSC are immune evasive and not immune privileged, they can only extend the survival of the co-transplanted iPSC-derived cardiomyocytes without preventing their ultimate disappearance (Yoshida et al., 2020).

#### Arrhythmias

A major concern raised by non-human primate studies in which ESC-derived cardiomyocytes have been transplanted has been the occurrence of ventricular arrhythmias (Chong et al., 2014; Shiba et al., 2016; Liu Y.-W. et al., 2018; Romagnuolo et al., 2019), some of which were life-threatening. Different mechanisms have been hypothesized but rather then re-entry induced by tracks of slow conduction, it seems that focal activation at the graft/host interface is a key trigger of these events (Liu Y.-W. et al., 2018; Romagnuolo et al., 2019). Of note, arrhythmias have usually been observed early, i.e., during the first post-transplantation weeks with a progressive diminution onward which could reflect a progressive *in situ* maturation of the graft toward a ventricular-like pattern. This supports the transplantation of a homogeneous population of cells featuring a mature electrophysiological phenotype but also emphasizes the importance of ensuring that the graft is appropriately purged from all cells which could still feature a pace-maker phenotype.

In an attempt to circumvent these issues while still targeting remuscularization of the diseased heart, other strategies have been developed which overall try either to reset the clock by restoring the ability of endogenous cardiomyocytes to divide or to convert host fibroblasts into new cardiomyocytes (reviewed in Sadek and Olson, 2020). The discussion of these strategies is beyond the scope of this paper but the important point is that a cell-induced improvement of function, which is the ultimate main objective, is not automatically correlated with an increase of the contractile cell pool. This has been well demonstrated in a mouse model of myocardial infarction where transplanted cardiovascular progenitor cells expressing a doxycycline-inducible *MYC* construct were shown to proliferate in the myocardium and then, following doxycycline removal, to differentiate in cardiomyocytes, endothelial and smooth muscle cells, leading to the formation of large grafts from the transplanted cells but without improvement of the contractile function (even though fibrosis was reduced) (Schwach et al., 2020). These data are in line with those published more than 10 years ago by van Laake et al. (2009) who had shown, in a mouse model, that post-infarction cardiac function was not improved by increasing graft size but rather seemed to be largely contributed by augmentation of the vascular density. Put together, these results are thus suggestive that other mechanisms than generation of new cardiomyocytes are involved in cell-induced improvement of outcomes, thereby lending support to the paracrine hypothesis.

### The Paracrine Hypothesis

Aside from “remuscularization,” a second mechanism whereby PSC-derived cardiac cells could act is paracrine signaling, i.e., the release of factors harnessing endogenous repair pathways (Garbern and Lee, 2013).

#### Evidence for a Paracrine Mechanism of Action

This hypothesis is strongly supported by three lines of reasoning. First, there is a consistent temporal discrepancy between the physical presence of cells in the transplanted tissue and the functional outcomes, i.e., an improvement in heart function is commonly demonstrated at a time where all grafted cells have disappeared. This applies to PSC cardiac derivatives as well. Thus, Riegler et al. (2015) found no difference in cardiac function between chronically infarcted rats transplanted with human ESC-derived cardiomyocytes mixed with collagen and those in which cells had previously been made non-viable by irradiation, thereby suggesting that cell engraftment was not directly responsible for functional improvements. Likewise, differences in ESC graft size have been shown not to translate into differences in the preservation of post-infarction heart function which further supports the idea that remuscularization may not be the key driver of the cell-associated therapeutic benefit (van Laake et al., 2009; Luo et al., 2014). More recently, ESC-derived cardiovascular progenitor cells were reported to improve function of infarcted and adequately immunosuppressed non-human primates at a time where cells are no longer detectable (Zhu et al., 2018). Admittedly, another macaque study has reported the persistence of substantial grafts until 3 months after transplantation, possibly because of the use of more differentiated cardiomyocytes, but the scarcity of their connexin-43 expression still makes uncertain a functionally effective coupling with host cardiomyocytes (Liu Y.-W. et al., 2018) and this concern is strengthened by the finding of scar tissue isolating ESC- and iPSC-cardiomyocyte grafts from the host myocardium of infarcted rats, which makes unlikely their direct contribution to cardiac contractility (van Laake et al., 2009; Lee et al., 2017). Second, cells are known to release a myriad of cytokines, growth factors and other biologics, many of them being packaged in extracellular vesicles (exosomes and microparticles) which can modulate the function of recipient cells through the delivery of their cargo (Patil et al., 2019) and this mechanism also pertains to vesicles released by PSC (Yuan et al., 2009). Extracellular vesicles can then shift recipient cell signaling pathways toward cardiac repair through multiple mechanisms, primarily mitigation of apoptosis, inflammation and fibrosis and stimulation of angiogenesis (Garikipati et al., 2018; Zhao et al., 2019), while the re-induction of a mitotic cycle of native cardiomyocytes leading to an increased contractile cell pool remains more controversial. Third, the extracellular vesicle-enriched secretome of stem cell-derived cardiac cells (or of MSC) recapitulates (and even sometimes outweighs) the beneficial effects of their parental cells an observation which has now been made across a wide variety of preclinical cardiac (Kervadec et al., 2016; Adamiak et al., 2018; El Harane et al., 2018; Rogers et al., 2019) and non-cardiac disease models (reviewed in Desgres and Menasché, 2019) and is consistent with the finding of an overlap of the microRNA profiles between iPSC-cardiomyocytes (Santoso et al., 2020) or MSC (Shao et al., 2017) and their respective exosomes.

Relying on a paracrine mechanism of action implies that cells behave as platforms releasing bioactive molecules and will remain only transiently in the grafted tissue; however, as nobody still knows exactly how long is enough for the cells to release the factors underpinning their therapeutic benefits, the use of adjunctive biomaterials to extend their residency time can still be justified (Chen et al., 2018; Waters et al., 2018; Han et al., 2019). Importantly, rejection does not need to be prevented any longer; it should only be delayed, which implies a short-duration of immunosuppression and consequently reduces the risk of drug-induced side effects. We previously mentioned that in the ESCORT trial, immunosuppressive drugs were only given for 1 month with good tolerance and the Japanese trial entailing the use of iPSC-derived cardiomyocyte cell sheets has likewise planned a limited period (3 months) of immunosuppression.

#### Use of the PSC Secretome

##### Advantages

The assumption that most, if not all, of the cardioprotective effects of stem cells can be duplicated by the exclusive use of their secretome has logically led to consider the latter as the only therapeutics that could be delivered. This approach has distinct clinically relevant advantages, including (1) a standardized pharma-like manufacturing process, (2) a likely lack of immunogenicity, provided the cells of origin themselves express little, if any, of the HLA I and II, which is the case for iPSC-derived cardiovascular progenitors (Lima Correa et al., 2020), and (3) a functional stability under cryo-storage compatible with an off-the-shelf availability. However, these presumed benefits have to be compounded by the still limited number of large animal proof-of-concept studies (Timmers et al., 2011; Gallet et al., 2017) and the absence, so far, of clinical trials allowing a thorough assessment of this approach in the context of heart failure.

##### Challenges

Regardless of the cautionary words above, the translational use of the secretome requires to address at least four main challenges, the first of which is the choice of the parental cells. Although the RNA and protein cargo of undifferentiated ESC (Khan et al., 2015) and iPSC (Bobis-Wozowicz et al., 2015) has shown cardio-protective effects both *in vitro* and in animal models of myocardial infarction, the clinical use of such a secretome would likely raise safety issues because of its possible enrichment with pluripotency markers (Ratajczak et al., 2006), which rather leads to privilege more differentiated cells as the source material. In this context, and in parallel to what has been shown for cells, the bioactivity of the secretome of cardiac-committed cells outweighs that of non-cardiac cells (Barile et al., 2018), possibly because the released factors are better tailored to the specific biology of the target cardiac tissue; even within the cardiac cell population, the secretome released by early progenitor cells seems to feature superior cardioprotective properties (Agarwal et al., 2017; Könemann et al., 2019) and these data are in line with our observations (El Harane et al., 2018) and those of others (Malik et al., 2013) that untreated adult cardiomyocytes secrete minimal amounts of exosomes. These findings strongly rationalize the choice of PSC as the parental cells because of the dual possibility of differentiating them into cardiac-committed cells and stopping the differentiation process at an early developmental stage. For this reason, our next clinical trial will entail the administration of an extracellular vesicle-enriched secretome of iPSC-derived cardiovascular progenitor cells (El Harane et al., 2018). A second challenge is the mode of delivery of the secretome. If direct intramyocardial injections are considered, a one-shot delivery without any additional carrier exposes to a rapid wash-out and an attendant loss of efficacy. Thus, the incorporation of the cell-produced biologics into biomaterials enabling a sustained release (Chen et al., 2018; Waters et al., 2018), much like for traditional stem cell therapy, can be an effective means of extending their retention and improving outcomes and this concept applies to PSC-derived cardiac cells (Liu B. et al., 2018). Here, a distinct advantage of cell-derived biologics is that they can be successfully embedded into scaffolds suitable for long-term cryopreservation and immediate availability, thereby overcoming survival and storage issues associated with the use of cells (Huang et al., 2020). However, a more appealing means of leveraging the acellular nature of the secretome is to deliver it intravenously. Although a limited amount of the injectate may then reach the target organ, the intravenous route is yet associated with a functional benefit which has been demonstrated across a wide variety of preclinical models of acute myocardial infarction (Timmers et al., 2011; Ciullo et al., 2019), Duchenne (Rogers et al., 2019) and chemotherapy-induced (Singla et al., 2012; Vandergriff et al., 2015; Sun et al., 2018; Milano et al., 2019) cardiomyopathies, as well as in non-cardiac disease models. One postulated mechanism of action of these intravenously injected EV-enriched secretomes is that following their predominant uptake by macrophages (Morishita et al., 2017) and liver sequestration (Wiklander et al., 2015), they could act like cells through a systemic modulation of inflammation triggering tissue-protective signals which are then conveyed to the target organ by the circulating cells (Walker et al., 2012). It is clear that the intravenous route has clinically relevant advantages: it is simple as it does not require costly equipment and facilities; more importantly, it is non-invasive and as such allows repeated administrations, which could be critical for optimizing the therapeutic benefit, as suggested by the finding that repeated administrations of transplanted cells are superior to the single administration of an equivalent cumulative dose (Tang et al., 2018). Of note, while all modes of cells/cell products delivery target the same objective, i.e., an improvement in heart function along with a reversal of adverse ventricular remodeling, they might conceivably reach this goal through different mechanisms of action, i.e., the predominant replenishment of the contractile cell pool along with an increased angiogenesis in the case of direct intramyocardial cardiac cell implantation as opposed to a systemic regulation of inflammation following intravenous infusions.

The last two translational challenges raised by the use of the cellular secretome are its degree and method of purification as well as the characterization of its content. However, these issues are not specific for PSC used as the parental cells and their discussion is beyond the scope of this chapter (for reviews, see Andriolo et al., 2018; Théry et al., 2018).

### A Common Mechanism-Independent Issue: Safety

Regardless of the putative mechanism of action, safety is obviously a key issue because the presence in the cell product of still undifferentiated and/or transformed cells could lead to their uncontrolled proliferation and give rise to a teratoma or a terato-carcinoma. The opponents of ESC have obviously rushed on this risk to demonize ESC (Wysoczynski and Bolli, 2020) in contempt of the actual data which show that although the first five patients to receive ESC derivatives, in this case oligodendrocytes for spinal cord injury, are now at 8 years of their treatment without any warning signal (Press release from Asterias Biotherapeutics Inc., January 24, 2019). Likewise, the first patient of the ESCORT trial has now a more than 5 year-follow up and is doing well without any abnormality on imaging studies (computerized tomography and ^18^F-FDG positron emission tomography scans). Nevertheless, it is obviously critical to purify the differentiated cells to ensure that they are no longer contaminated with unwanted and still pluripotent cells.

Multiple purification methods have been developed (reviewed in Ban et al., 2017; Moon et al., 2017), of which only a sorting based on surface markers is currently suitable for large-scale GMP-compliant applications. In the ESCORT trial, we used SSEA-1 as a marker for cells which were no longer undifferentiated and used magnetic bead-bound anti-SSEA-1 antibodies for selecting them. Even though this mode of immunomagnetic positive selection has been used for years for enriching in hematopoietic cell populations, a negative mode of selection targeting markers for pluripotency (Tang et al., 2011) is more appealing in that it would allow the final cell therapy product for patient use to remain magnetic bead-free. Other strategies based on microfluidics or field-flow fractionation are currently investigated and might be of interest if they can be scaled-up under GMP conditions. Of note, selection of the cardiac-committed cells is primarily relevant to the use of cardiovascular progenitors where stage-specific markers have to be used (*Isl-1* in the ESCORT trial) since an extended period of culture is expected to yield an almost pure population of cardiomyocytes whose identity can be confirmed by a battery of markers (Flk-1, PDGFR- α, signal-regulatory protein alpha [(SIRPA)] and vascular cell adhesion molecule 1 (VCAM1)] and which, in principle, does not harbor undifferentiated cells any longer.

However, regardless of the cell culture strategy, it is mandatory to ensure that the final product which will be delivered to the patient has no tumorigenic potential. This first implies *in vitro* checking for its genetic stability to eliminate the occurrence of cancer-driving mutations that can be induced by culture conditions and time in culture (Goldring et al., 2011; Oliveira et al., 2014). Several quality control metrics have been proposed to ensure that oncogenic networks have not been activated (reviewed in Assou et al., 2018). As an example, the cardiovascular SSEA-1^+^ cells used in the ESCORT trial were assessed by karyotyping, fluorescence *in situ* hybridization (FISH) and array comparative genomic hybridization (aCGH). Of note, iPSC may be more prone to genetic/epigenetic alterations than ESC because of the potential of mutations already present in the parental cells from which they are reprogrammed (Ben-David and Benvenisty, 2011; Oliveira et al., 2014), with the caveat that it can be difficult to establish a correlation between genetic modifications of the cells and the actual risk that will form a tumor *in vivo*. Maybe, in the future, the organs-on-chip technology will provide a human-like micro-environment more suitable for an accurate risk prediction. The identity of the cardiac-committed product also needs to be tested by lineage-specific markers, usually by flow cytometry and/or gene sequencing, in particular to ensure that pluripotency genes have been appropriately knocked down. It may also be advisable to enrich the risk analysis with assays evaluating the potential of the cells to cause genotoxicity and chromosomal damage.

Despite the utility of these *in vitro* tests, tumorigenicity studies in animals still represent a major pillar of the preclinical safety assessment. Several factors have then to be taken into consideration, which include the number of cells to be inoculated, the duration of follow-up, the site of transplantation and the limit of detection of the relevant assays (Sato et al., 2019). The choice of the animal model is particularly critical, keeping in mind that most studies entail transplanting human cells into rodents and, as such, should be interpreted cautiously since xenotransplantation is less likely to induce teratomas than allografts (Erdö et al., 2003). This has been the main argument for us to test Rhesus ESC-derived cardiovascular progenitors in infarcted Rhesus monkeys with the premise that such an intraspecies transplantation would sensitize the potential occurrence of a tumor (Blin et al., 2010). Importantly, these teratoma experiments should include spiking experiments with different ratios of undifferentiated/lineage-committed cells to identify the threshold above which a tumor occurs and thus helps defining lot release criteria (Priest et al., 2015). This information is a critical component of the risk assessment. Finally, off-target adverse effects have to be ruled out by biodistribution studies.

## Perspectives

### Remuscularization

For those who remain committed to use PSC-derived cardiac-committed cells as physical and expectedly permanent substitutes for the lost cardiomyocytes, there are at least three areas of research which need to be prioritized. The first is to define the optimal stage of differentiation at which cells should be transplanted. As mentioned above, there are no robust arguments favoring early progenitors versus more mature cardiomyocytes but, assuming that the latter could more efficiently increase pump function, it is mandatory to address the risk of arrhythmias by optimizing the maturation state of the transplanted cardiomyocytes and thus mitigate an electrical mismatch at the graft/host interface (Romagnuolo et al., 2019); this can be achieved by a variety of strategies including co-culture with endothelial cells, prolongation of the culture time, control of the composition, topography and stiffness of the extracellular matrix, mechanical and electrical stimulation (Le and Hasegawa, 2019). The second area of research pertains to the development of strategies allowing an immune tolerance of these allogeneic cells in order to avoid the use of high doses of immunosuppressive drugs and their cohort of adverse effects. In this setting, the use of HLA-haplotyped cell lines (Neofytou et al., 2015) could be helpful by allowing a better match between the grafted cells and the immune profile of the recipient and is clinically doable as HLA-matched cells are currently transplanted in a Parkinson trial: https://upload.umin.ac.jp/cgi-open-bin/ctr_e/ctr_view.cgi?recptno=R000038278. However, the most conceptually attractive option could be the use of gene editing to generate cell lines no longer expressing HLA-I and -II antigens but engineered to express molecules preventing cell destruction by Natural Killer cells (Xu H. et al., 2019), provided that the efficacy and safety of these “universal” cell lines can be validated. One step further, co-transplanting cardiomyocytes and dendritic cells derived from the *same* PSC line might induce a selective tolerance to T cells reactive to the alloantigens expressed by the PSC derivatives. In the context of these efforts to “lighten” immunosuppressive regimens, another approach, possibly easier to implement in the clinics, could be the use of regulatory T cells or even their derived exosomes which have been shown to successfully extend liver graft survival (Chen et al., 2019).

Finally, even though the success of pro-survival approaches could enable a prolonged engraftment of the cells, it is likely that their number will progressively decrease over time. This assumption is supported by the finding that cardiac function was significantly improved in mice injected with ESC-derived cardiomyocytes at 4 weeks after transplantation but that this benefit was not retained at 3 months (van Laake et al., 2007), thereby suggesting that maintenance of a therapeutic effect likely requires repeated administrations. In the clinics, this can only be achieved, easily and safely, by intravenous injections. Admittedly, however, intravenously injected ESC-derived endothelial cells or iPSC-derived neural stem cells have been therapeutically effective in ischemic hindlimb (Huang et al., 2010) and amyotrophic lateral sclerosis (Nizzardo et al., 2014) models, respectively, without causing short-term adverse events, but one could be concerned about the safety of a systemic delivery of PSC derivatives and it is uncertain that such a strategy would be approved by the regulators (it might be the reason why the Chinese trial mentioned above and which had planned intravenous infusions of iPSC-derived cardiomyocytes in 3 heart failure patients has not yet started 18 months after its official registration). In this context, an innovative approach has been proposed which consists of implanting an epicardial device connected to a subcutaneous port which can be periodically replenished with. The extent to which this system, which has shown promising results in a rodent model of myocardial infarction (Whyte et al., 2018), can be translated clinically remains to be established.

### Paracrine Signaling

Reliance on a paracrine mechanism of action logically leads to consider to exclusively deliver the cellular secretome even though its effects on *in vitro* potency assays may not be representative of what happens when it is directly released by the parental cells *in vivo*. This limitation, however, may not be so relevant as demonstrated almost 10 years ago by Timmers et al. (2011) who showed, in a pig model of myocardial infarction, the protective effects of the intravenously infused conditioned medium (only clarified and concentrated) of ESC-derived MSC. Further confirmation has been brought more recently in a rat model of myocardial infarction in which a single intramyocardial injection of the total conditioned media released by neonatal human CSC was significantly more effective functionally than either the parental cells of their purified exosomal fraction (Sharma et al., 2017). Nevertheless, one area of research is to identify the key drivers of the biological effects of the cellular secretome and many different miRNAs and proteins have been reported as the most effective candidates. While it is tempting to identify a short list of compounds in the perspective of synthetizing them as a multi-targeted drug, it could indeed be counter-productive to deconstruct the cargo of the secretome because of the multiplicity of its components and their possible interactions leading to synergistic effects, notwithstanding the challenge of artificially recapitulating a complex repertoire of bioactive molecules. Another reason for keeping the whole extracellular vesicle payload intact is that it can be shuttled in the recipient cells without the risk of degradation because of the protection afforded by the lipid bilayer of the vesicles whereas it is unlikely it would still be the case if its individual components had to be delivered individually. A second area of research pertains to the means of “priming” the parental cells during the culture period to enrich the extracellular vesicle package with proteins and other biologics that can bolster their biological effects (Wiklander et al., 2019; Xu R. et al., 2019; Yuan et al., 2019). A third clinically relevant perspective pertains to the optimized delivery of the cellular secretome. Although using source cells which share the same repertoire of surface receptors as those of the target tissue can yet be therapeutically beneficial, as discussed above, a greater benefit could likely be yielded by a more selective targeting of the injectate toward the heart, which can be achieved by two different approaches. The first consists of engineering the parental cells to make them overexpressing receptors expected to traffic to their extracellular vesicles and whose display on the external part of the vesicular membrane can enhance recognition by target cells, docking and vesicle uptake. This strategy has been shown successful for promoting homing of intravenously injected exosomes derived from cardiac progenitor cells overexpressing CXCR4 to enhance interaction of this receptor with its SDF-1α ligand which is upregulated in ischemic tissues (Ciullo et al., 2019). This approach, however, is challenged by the risk of peptide degradation (Hung and Leonard, 2015), hence the interest of an alternate approach based on the direct engineering of the secreted vesicles by anchorage of cardiac-specific peptides to their membrane (Vandergriff et al., 2018) or modifications of their glysosylation pattern to promote binding to endothelial selectins (Sackstein, 2009; Lo et al., 2016). A last clinically relevant issue is dosing. There is consistent evidence that the effects of extracellular vesicles are dose-dependent (Kervadec et al., 2016) while the apparently low number of miRNA carried by exosomes (Chevillet et al., 2014) likely requires large amounts of them to be transferred to convey a significant biological effect, particularly following a systemic delivery. Different metrics can be considered here such as an absolute number of particles, a protein concentration, the vesicle number to protein ratio or a “cell equivalent,” i.e., the amount of particles assumed to be released by a given number of the parental cells but no consensus has emerged yet.

In conclusion, while iPSC look more attractive for disease modeling and drug screening, both ESC and iPSC share the same potential for inducing heart repair and possibly regeneration. For reasons related to practicality, iPSC-derived cardiac-committed cells, regardless of their stage of differentiation, may provide a better cost-effective model, particularly if they are primarily used as *ex vivo* biofactories producing a biologically active secretome which would then represent the therapeutics delivered to the patient. This approach would allow to leverage the cardioprotective potential of PSC derivatives while overcoming the safety issues that might be associated with their direct delivery. Future clinical studies will tell whether such an expectation can be really met. In the meantime, the observation that by the end of 2019, there were at least 54 registered trials testing PSC derivatives (Kobold et al., 2020) provides compelling evidence that regardless of the cell source (ESC or iPSC), the clinical indication and the delivery strategy, PSC-derived lineage-specific cells have now integrated the armamentarium of therapies against a wide variety of diseases.

## Author Contributions

PM wrote the manuscript.

## Conflict of Interest

The author declares that the research was conducted in the absence of any commercial or financial relationships that could be construed as a potential conflict of interest.

## Footnotes

1 News at a glance. *Science* 362, 268–270. doi: 10.1126/science.362.6412.268

2 https://www.japantimes.co.jp/news/2020/01/28/national/science-health/osaka-university-transplants-ips-cell-based-heart-cells-worlds-first-clinical-trial/

3 https://www.umin.ac.jp/ctr/
